# V-Spline: An Adaptive Smoothing Spline for Trajectory Reconstruction

**DOI:** 10.3390/s21093215

**Published:** 2021-05-06

**Authors:** Zhanglong Cao, David Bryant, Timothy C.A. Molteno, Colin Fox, Matthew Parry

**Affiliations:** 1 SAGI West, School of Molecular and Life Sciences, Curtin University, Perth 6085, Australia; zhanglong.cao@curtin.edu.au; 2 Department of Mathematics & Statistics, University of Otago, Dunedin 9054, New Zealand; david.bryant@otago.ac.nz; 3 Department of Physics, University of Otago, Dunedin 9054, New Zealand; tim@elec.ac.nz (T.C.A.M.); colin.fox@otago.ac.nz (C.F.)

**Keywords:** hermite spline basis functions, cross-validation, adaptive penalty, piecewise continuous

## Abstract

Trajectory reconstruction is the process of inferring the path of a moving object between successive observations. In this paper, we propose a smoothing spline—which we name the V-spline—that incorporates position and velocity information and a penalty term that controls acceleration. We introduce an adaptive V-spline designed to control the impact of irregularly sampled observations and noisy velocity measurements. A cross-validation scheme for estimating the V-spline parameters is proposed, and, in simulation studies, the V-spline shows superior performance to existing methods. Finally, an application of the V-spline to vehicle trajectory reconstruction in two dimensions is given, in which the penalty term is allowed to further depend on known operational characteristics of the vehicle.

## 1. Introduction

Global Positioning System (GPS) technology has become an essential tool in a wide range of applications involving moving vehicles, from transport management [1] and traffic safety studies [2] to modern precision farming [3]. Nevertheless, the accuracy of GPS tracking seems to be neglected in many applications [4,5]. Even if an accurate GPS device is utilized, GPS remains subject to various systematic errors due to the number of satellites in view, uncertainty in satellite orbits, clock and receiver issues, etc. [6,7]. These measurements are usually irregularly recorded, leading to what is known as irregularly spaced or intermittent data. Reconstruction or forecasting based on irregularly spaced data is usually more complicated and less accurate than that based on regularly spaced data [8].

To be fit for purpose, trajectory reconstruction must be accurate and robust. Two key issues for reconstruction are (i) how to handle observations that are inherently noisy measurements of the truth, and (ii) how to interpolate appropriately between observations, also known as path interpolation. In this context, statistical smoothing techniques can be useful processing tools because they are designed to minimize the impact of random error, and still typically require less time to detect random errors than visual inspection [9].

It has been shown previously that, if kinematic information such as velocity and acceleration can be included, interpolation and hence trajectory reconstruction can be greatly improved. The authors in [10,11] used B-splines to give a closed-form expression for a trajectory with continuous second derivatives that passes through the position points smoothly while ignoring outliers. The authors in [12] presented a quintic spline trajectory reconstruction algorithm connecting a series of reference knots that produces continuous position, velocity, and acceleration profiles in the context of computer (or computerized) numerical control (CNC). The authors in [13] gave a piecewise cubic reconstruction found by matching the observed position and velocity at the endpoints of each interval; this is essentially a Hermite spline. The authors in [14] also used Hermite interpolation to fit position, velocity and acceleration with given kinematic constraints. The authors in [15] implemented spline-based trajectories in order to overcome parametric singularities that occur in some reconstruction methods. The author in [16] proposed the kinematic interpolation approach that uses a set of kinematic equations to describe the motion of an object in terms of polynomial splines. Based on an adaptive cubic spline interpolation, the authors in [17] proposed an approach that, in the context of the Aircraft Communication Addressing and Reporting System, improves the smoothness and precision of trajectory reconstruction.

These approaches focus on optimal paths that are typically the shortest in either distance or time between starting and end points. Additionally, in some approaches, the moving object is assumed to be a point-like object. In this case, the object can rotate about itself to orient along the path in the direction of the goal point [15]. This assumption is unlikely to be appropriate for a real vehicle or vessel, particularly a tractor, which is the motivating example in our study.

Modern farming relies on the precise application of fertilizers, pesticides and irrigation. Large commercial farms typically operate a fleet of farm vehicles for these tasks, and it is of crucial importance for economic, environmental and regulatory reasons that the location and operational characteristics of these vehicles are recorded systematically and accurately. In order to do this, it is becoming standard to equip farm vehicles with GPS units to record the location of the vehicle on the farm. It is the goal of this study to develop an appropriate tool to reconstruct vehicle trajectories from such data, particularly when it is intermittent and noisy.

In this study, we assume that we have independent records of the position and velocity of a moving object at a sequence of observation times. Traditional methods often assume motion with constant speed between two observations times, but this will not work well in our case. Additionally, motivated by the fact that tractors often work in open fields, we assume no further information is available to constrain the position of the object. Initially, we constructed trajectories in terms of a *Hermite cubic spline* basis [18,19]. In each interval, the reconstruction is clearly continuous, as are its first and second derivatives. The goal is then to connect the piecewise splines keeping the trajectory and its first derivative continuous at the interior knots. In this approach, the trajectory is not required to pass through each knot and the main objective is the smoothness of the path, not a shortest or minimum-time path. To formalize this procedure, we propose a new objective function that incorporates velocity information and includes an adaptive penalty term. The penalty term utilises information about the distance and travel time on each interval. We dub the proposed smoothing spline the V-spline because it incorporates velocity information and can be applied to vehicle and vessel tracking. We show that the V-spline works better than other methods in simulation studies and that it produces satisfactory outcomes in a real-world application.

The structure of this paper is as follows: in Section 2, we introduce the basis functions and the V-spline objective function that depends both on position residuals $y_{i}-f\left(t_{i}\right)$ and velocity residuals $v_{i}-f^{\prime }\left(t_{i}\right)$. A new parameter γ in the objective function controls the degree to which the velocity information is used in the reconstruction. We show that the V-spline can be written in terms of modified Hermite spline basis functions. We also introduce a particular adaptive V-spline that seeks to control the impact of irregularly sampled observations and noisy velocity measurements. In Section 3, a cross-validation scheme for estimating the V-spline parameters is given. Section 4 details the performance of the V-spline on simulated data based on the *Blocks*, *Bumps*, *HeaviSine* and *Doppler* test signals [20]. Finally, an application of the V-spline to a two-dimensional data set is presented in Section 5. R code for implementing V-spline and reproducing our outcomes is provided as Appendix A, Appendix B and Appendix C at the end of the manuscript.

## 2. V-Spline

### 2.1. Objective Function

Conventional smoothing spline estimates of f(t) appear as a solution to the following minimization problem: find $\hat{f}\in C^{\left(2\right)}\left[a,b\right]$ that minimizes the penalized residual sum of squares,

(1) $$\mathrm{RSS}=\sum_{i=1}^{n}{\left(y_{i}-f\left(t_{i}\right)\right)}^{2}+\lambda \int_{a}^{b}{\left(f^{\prime \prime }\left(t\right)\right)}^{2}dt$$

for a pre-specified value λ>0 [21,22,23]. The objective function combines goodness-of-fit to the data with a measure of roughness [24].

For V-splines, we consider the situation of paired position data $y=\left\{y_{1},\ldots ,y_{n}\right\}$ and velocity data $v=\left\{v_{1},\ldots ,v_{n}\right\}$ at a sequence of times satisfying $a=t_{0}\leq t_{1}<t_{2}<\cdots <t_{n}\leq t_{n+1}=b$. For $f\in C_{p.w.}^{\left(2\right)}\left[a,b\right]$, where the second derivative of f(t) is piecewise continuous, we define the objective function

(2) $$J\left[f\right]=\frac{1}{n}\sum_{i=1}^{n}{\left(y_{i}-f\left(t_{i}\right)\right)}^{2}+\frac{\gamma }{n}\sum_{i=1}^{n}{\left(v_{i}-f^{\prime }\left(t_{i}\right)\right)}^{2}+\int_{a}^{b}\lambda \left(t\right){\left(f^{\prime \prime }\left(t\right)\right)}^{2}dt,$$

where γ>0, and we have chosen the penalty function λ(t) to be a piecewise constant function on interior intervals, i.e for $t\in [t_{i},t_{i+1}),i=1,\ldots ,n-1$,

(3) $$\lambda \left(t\right)=\lambda_{i}.$$

In fact, each $f_{i}\in C_{\left[t_{i},t_{i+1}\right]}^{\left(2\right)}$ is a Hermite spline which satisfies the properties of a cubic spline. The complete spline function *f*, which connects all $f_{i}$s, has piecewise continuous second derivative, and will be continuous if a particular condition is met. The second derivative $f^{\prime \prime }$ is zero on the exterior intervals $\left[a,t_{1}\right]$ and $\left[t_{n},b\right]$. From now on, we will understand λ(t) to be piecewise constant (3), and we will often use λ to refer to the set of $\lambda_{i}$.

**Theorem** **1.** *For n≥2, the objective function J[f] is uniquely minimized by a V-spline, piecewise on the intervals $\left[t_{i},t_{i+1}\right)$, i=1,…,n−1, and linear on $\left[a,t_{1}\right]$ and $\left[t_{n},b\right]$.*

The proof of Theorem 1 is in Appendix B.

*Remark:* In the language of splines, the points $t_{1},\ldots ,t_{n}$ are the interior knots of the V-spline, and $a=t_{0},b=t_{n+1}$ are the exterior or boundary knots.

### 2.2. Basis Functions

The cubic Hermite spline $f^{\left(i\right)}\left(t\right)$ on an arbitrary interval $\left[t_{i},t_{i+1}\right)$ with two consecutive points $\left\{y_{i},v_{i}\right\}$ and $\left\{y_{i+1},v_{i+1}\right\}$ is expressed as

(4) $$f^{\left(i\right)}\left(t\right)=h_{00}^{\left(i\right)}\left(t\right)y_{i}+h_{10}^{\left(i\right)}\left(t\right)v_{i}+h_{01}^{\left(i\right)}\left(t\right)y_{i+1}+h_{11}^{\left(i\right)}\left(t\right)v_{i+1},$$

where the basis functions are

(5) $$\begin{array}{cc} \; & h_{00}^{\left(i\right)}\left(t\right)=\left\{\begin{array}{cc} 2{\left(\frac{t-t_{i}}{t_{i+1}-t_{i}}\right)}^{3}-3{\left(\frac{t-t_{i}}{t_{i+1}-t_{i}}\right)}^{2}+1 & t_{i}\leq t<t_{i+1}\\ 0 & \mathrm{otherwise} \end{array}\right., \end{array}$$

(6) h10it=t−ti3ti+1−ti2−2t−ti2ti+1−ti+t−ti.ti≤t<ti+10otherwise,

(7) h01it=−2t−titi+1−ti3+3t−titi+1−ti2+ti≤t<ti+10otherwise,

(8) h11it=t−ti3ti+1−ti2−t−ti2ti+1−ti+t−+123−ti≤t<ti+10otherwise.

For V-splines, a slightly more convenient basis is given by ${\left\{N_{k}\left(t\right)\right\}}_{k=1}^{2n}$, where $N_{1}\left(t\right)=h_{00}^{\left(1\right)}\left(t\right)$, $N_{2}\left(t\right)=h_{10}^{\left(1\right)}\left(t\right)$, and for all i=1,2,…,n−2, we have

$$\begin{array}{cc} N_{2i+1}\left(t\right) & =h_{01}^{\left(i\right)}\left(t\right)+h_{00}^{\left(i+1\right)}\left(t\right),\\ N_{2i+2}\left(t\right) & =h_{11}^{\left(i\right)}\left(t\right)+h_{10}^{\left(i+1\right)}\left(t\right), \end{array}$$

and

$$\begin{array}{cc} N_{2n-1}\left(t\right) & =\left\{\begin{array}{cc} h_{01}^{\left(n-1\right)}\left(t\right) & \mathrm{if}\;t<t_{n}\\ 1 & \mathrm{if}\;t=t_{n} \end{array}\right.,\\ N_{2n}\left(t\right) & =h_{11}^{\left(n-1\right)}\left(t\right). \end{array}$$

Therefore, any $f\in C_{p.w.}^{\left(2\right)}\left[a,b\right]$ can then be represented in the form

(9) $$f\left(t\right)=\sum_{k=1}^{2n}N_{k}\left(t\right)\theta_{k},$$

where ${\left\{\theta_{k}\right\}}_{k=1}^{2n}$ are parameters corresponding with the “true” position $f(t_{i})$ and velocity $f^{\prime }\left(t_{i}\right)$ at the observation points.

### 2.3. Computing the V-Spline

In terms of the basis functions in the previous section, the objective function (2) is given by

(10) $$nJ\left[f\right]\left(\theta ,\lambda ,\gamma \right)={\left(y-B\theta \right)}^{⊤}\left(y-B\theta \right)+\gamma {\left(v-C\theta \right)}^{⊤}\left(v-C\theta \right)+n\theta^{⊤}\Omega_{\lambda }\theta ,$$

where *B* and *C* are n×2n matrices with components

(11) $$\begin{array}{cc} {\left[B\right]}_{ij} & =N_{j}\left(t_{i}\right)=\left\{\begin{array}{cc} 1, & j=2i-1\\ 0, & \mathrm{otherwise} \end{array}\right. \end{array}$$

(12) $$\begin{array}{cc} {\left[C\right]}_{ij} & =N_{j}^{\prime }\left(t_{i}\right)=\left\{\begin{array}{cc} 1, & j=2i\\ 0, & \mathrm{otherwise} \end{array}\right. \end{array}$$

and $\Omega {}_{\lambda }$ is a 2n×2n matrix with components ${\left[\Omega_{\lambda }\right]}_{jk}=\int_{a}^{b}\lambda \left(t\right)N_{j}^{\prime \prime }\left(t\right)N_{k}^{\prime \prime }\left(t\right)dt$. In the following, we reserve the use of boldface for n×1 vectors and n×n matrices.

The detailed structure of $\Omega {}_{\lambda }$ is presented in Appendix A. It is convenient to write $\Omega_{\lambda }=\sum_{i=1}^{n-1}\lambda_{i}\Omega^{\left(i\right)}$, where ${\left[\Omega^{\left(i\right)}\right]}_{jk}=\int_{t_{i}}^{t_{i+1}}N_{j}^{\prime \prime }\left(t\right)N_{k}^{\prime \prime }\left(t\right)dt$. It is then evident that $\Omega {}_{\lambda }$ is a bandwidth four matrix.

Since Equation (10) is a quadratic form in terms of θ, it is straightforward to establish that the objective function is minimized at

(13) $$\hat{\theta }={\left(B^{⊤}B+\gamma C^{⊤}C+n\Omega_{\lambda }\right)}^{-1}\left(B^{⊤}y+\gamma C^{⊤}v\right),$$

which can be identified as a generalized ridge regression. The fitted V-spline is then given by $\hat{f}\left(t\right)=\sum_{k=1}^{2n}N_{k}\left(t\right){\hat{\theta }}_{k}$.

The V-spline is an example of a linear smoother [25]. This is because the estimated parameters in Equation (13) are a linear combination of y and v. Denoting by $\hat{f}$ and $\hat{f^{\prime }}$ the vector of fitted values $\hat{f}\left(t_{i}\right)$ and $\hat{f^{\prime }}\left(t_{i}\right)$ at the training points $t_{i}$, we have

(14) $$\begin{array}{ccc} \hat{f} & =B{\left(B^{⊤}B+\gamma C^{⊤}C+n\Omega_{\lambda }\right)}^{-1}\left(B^{⊤}y+\gamma C^{⊤}v\right) & :=S_{\lambda ,\gamma }y+\gamma T_{\lambda ,\gamma }v \end{array}$$

(15) $$\begin{array}{ccc} \hat{f^{\prime }} & =C{\left(B^{⊤}B+\gamma C^{⊤}C+n\Omega_{\lambda }\right)}^{-1}\left(B^{⊤}y+\gamma C^{⊤}v\right) & :=U_{\lambda ,\gamma }y+\gamma V_{\lambda ,\gamma }v \end{array}$$

where $S_{\lambda ,\gamma },T_{\lambda ,\gamma },U_{\lambda ,\gamma }$ and $V_{\lambda ,\gamma }$ are smoother matrices that depend only on $t_{i},\lambda \left(t\right)$ and γ. It is not hard to show that $S_{\lambda ,\gamma }$ and $V_{\lambda ,\gamma }$ are symmetric, positive semi-definite matrices. Note that $T_{\lambda ,\gamma }=U_{\lambda ,\gamma }^{⊤}$.

**Corollary** **1.** *If f(t) is a V-spline, then, for almost all y and v, $f^{\prime \prime }\left(t\right)$ is continuous at the knots if and only if γ=0 and $\lambda_{i}=\lambda_{0}$, for all i=1,…,n−1.*

### 2.4. Adaptive V-Spline

Until now, we have not explicitly considered the impact of irregularly sampled observations of noisy measurements of velocity on trajectory reconstruction. In order to do this, it is instructive to evaluate the contribution to the penalty term from the interval $\left[t_{i},t_{i+1}\right)$. Using (4), it is relatively straightforward to show that

(16) $${\hat{f}}^{\prime \prime }\left(t\right)=\frac{1}{t_{i+1}-t_{i}}\left\{6\left(\varepsilon_{i}^{+}+\varepsilon_{i}^{-}\right)\frac{t-t_{i}}{t_{i+1}-t_{i}}-2\left(2\varepsilon_{i}^{+}+\varepsilon_{i}^{-}\right)\right\},$$

where $\varepsilon_{i}^{+}=v_{i}-{\bar{v}}_{i}$, $\varepsilon_{i}^{-}=v_{i+1}-{\bar{v}}_{i}$ and ${\bar{v}}_{i}=\left(y_{i+1}-y_{i}\right)/\left(t_{i+1}-t_{i}\right)$ is the average velocity over the interval. The $\varepsilon_{i}^{\pm }$ can be interpreted as the difference at time $t_{i}$ and $t_{i+1}^{-}$ respectively between the velocity implied by an interpolating Hermite spline and the velocity implied by a straight line reconstruction.

The contribution to the penalty term is then

(17) $$4\lambda_{i}\frac{{\left(\varepsilon_{i}^{+}\right)}^{2}+\varepsilon_{i}^{+}\varepsilon_{i}^{-}+{\left(\varepsilon_{i}^{-}\right)}^{2}}{\Delta T_{i}},$$

where $\Delta T_{i}=t_{i+1}-t_{i}$. We call the quantity ${\left(\varepsilon_{i}^{+}\right)}^{2}+\varepsilon_{i}^{+}\varepsilon_{i}^{-}+{\left(\varepsilon_{i}^{-}\right)}^{2}$, the square of the *discrepancy* of the velocity on the interval $\left[t_{i},t_{i+1}\right)$.

**Figure 1:**
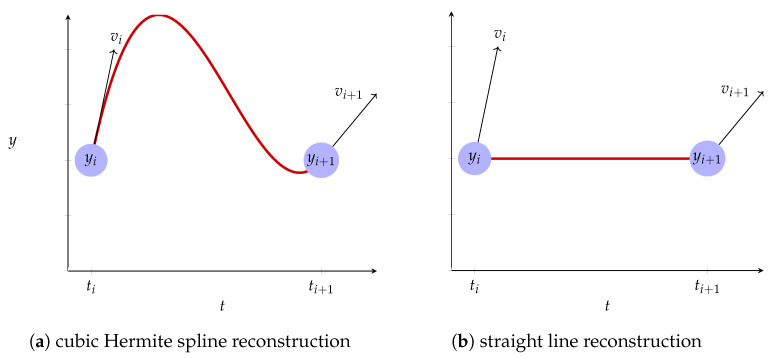
Comparing cubic Hermite spline reconstruction and straight line reconstruction. When $\Delta T_{i}=t_{i+1}-t_{i}$ is large or ${\bar{v}}_{i}\Delta T_{i}=y_{i+1}-y_{i}$ is small, the adaptive V-spline favours straighter reconstructions.

As a consequence of (17), larger time intervals will tend to contribute less to the penalty term (other things being equal). However, this is exactly when we would expect the velocity at the endpoints of the interval to provide less useful information about the trajectory over the interval. In the case when the observed change in position is small, i.e., when $y_{i+1}-y_{i}={\bar{v}}_{i}\Delta T_{i}\approx 0$, over-reliance on noisy measurements of velocity will result in “wiggly” reconstructions. In these two instances—graphically depicted in Figure 1a—we would like the V-spline to adapt and to favor straighter reconstructions; this is a deliberate design choice. We can achieve this by choosing

(18) $$\lambda_{i}=\eta \frac{\Delta T_{i}}{{\bar{v}}_{i}^{2}},$$

where η is a parameter to be estimated. The penalty term then takes a particularly compelling form: the contribution from the interval $\left[t_{i},t_{i+1}\right)$ (17) is proportional to

(19) $${\left(\frac{\mathrm{discrepancy in velocity}}{\mathrm{average velocity}}\right)}^{2}$$

for all *i*. We call the resulting spline the *adaptive* V-spline. The spline when $\lambda_{i}=\lambda_{0}$ or, more accurately, when $\lambda_{i}$ is independent of $\Delta T_{i}$ and ${\bar{v}}_{i}$, we call it the *non-adaptive* V-spline.

## 3. Parameter Selection and Cross-Validation

The issue of choosing the smoothing parameter is ubiquitous in curve estimation and there are two different philosophical approaches to the problem. The first is to regard the free choice of smoothing parameter as an advantageous feature of the procedure. The second is to let the data determine the parameter [22,26], using a procedure such as cross-validation (CV) or generalized cross-validation (GCV) [21]. We prefer the latter and use the data with GCV to train our model and find the best parameters.

In standard regression, which assumes the mean of the observation errors is zero, the true regression curve f(t) has the property that, if an observation $y_{i}$ is omitted at time point $t_{i}$, the value $f(t_{i})$ is the best predictor of $y_{i}$ in terms of mean squared error [22]. We use this observation to motivate a leave-one-out cross-validation scheme to estimate λ and γ for both the non-adaptive and the adaptive V-splines.

Let ${\hat{f}}^{\left(-i\right)}\left(t,\lambda ,\gamma \right)$ be the minimizer of

(20) $$\begin{array}{c} \frac{1}{n}\sum_{j\neq i}{\left(y_{j}-f\left(t_{j}\right)\right)}^{2}+\frac{\gamma }{n}\sum_{j\neq i}{\left(v_{j}-f^{\prime }\left(t_{j}\right)\right)}^{2}+\int_{a}^{b}\lambda \left(t\right){\left(f^{\prime \prime }\left(t\right)\right)}^{2}dt, \end{array}$$

and define the cross-validation score

(21) $$\begin{array}{c} \underset{\lambda ,\gamma >0}{arg min}CV\left(\lambda ,\gamma \right)\;=\;\underset{\lambda ,\gamma >0}{arg min}\sum_{i=1}^{n}{\left(y_{i}-{\hat{f}}^{\left(-i\right)}\left(t_{i},\lambda ,\gamma \right)\right)}^{2}. \end{array}$$

We then choose λ and γ that jointly minimize CV(λ,γ).

The following theorem establishes that we can compute the cross-validation score without knowing the ${\hat{f}}^{\left(-i\right)}\left(t,\lambda ,\gamma \right)$:

**Theorem** **2.** *The cross-validation score of a V-spline satisfies*

(22) $$\underset{\lambda ,\gamma >0}{arg min}CV\left(\lambda ,\gamma \right)=\underset{\lambda ,\gamma >0}{arg min}\sum_{i=1}^{n}{\left(\frac{y_{i}-\hat{f}\left(t_{i}\right)+\gamma \frac{T_{ii}}{1-\gamma V_{ii}}\left(v_{i}-{\hat{f}}^{\prime }\left(t_{i}\right)\right)}{1-S_{ii}-\gamma \frac{T_{ii}}{1-\gamma V_{ii}}U_{ii}}\right)}^{2},$$

*where $\hat{f}$ is the V-spline smoother calculated from the full data set with smoothing parameter λ and γ, and $S_{ii}={\left[S_{\lambda ,\gamma }\right]}_{ii}$, etc.*

The proof of Theorem 2 is in Appendix C.

## 4. Simulation Study

In this section, we give an extensive comparison of methods for equal-spaced data. The comparison is based on the ability to reconstruct trajectories derived from *Blocks*, *Bumps*, *HeaviSine* and *Doppler*, which were used in [20,27,28] to mimic problematic features in imaging, spectroscopy and other types of signal processing.

Letting g(t) denote any one of *Blocks*, *Bumps*, *HeaviSine* or *Doppler*, we treat g(t) as the instantaneous velocity of the trajectory f(t) at time *t*, i.e., $f^{\prime }\left(t\right)=g\left(t\right)$. Setting $f(t_{1})=0$, the position is then updated in terms of the average velocity over each interval:

(23) $$f\left(t_{i+1}\right)=f\left(t_{i}\right)+\frac{g\left(t_{i}\right)+g\left(t_{i+1}\right)}{2}\left(t_{i+1}-t_{i}\right),$$

which is accurate to the second order in $t_{i+1}-t_{i}$. Finally, the observed position and velocity are found by adding i.i.d. zero-mean Gaussian noise:

(24) $$\begin{array}{c} \begin{array}{cc} y_{i} & =f\left(t_{i}\right)+\varepsilon_{i}^{\left(f\right)},\\ v_{i} & =g\left(t_{i}\right)+\varepsilon_{i}^{\left(g\right)}, \end{array} \end{array}$$

where $\varepsilon_{i}^{\left(f\right)}∼N\left(0,\sigma_{f}/SNR\right)$, $\varepsilon_{i}^{\left(g\right)}∼N\left(0,\sigma_{g}/SNR\right)$, $\sigma_{f}$ is the standard deviation of the positions $f(t_{i})$, $\sigma_{g}$ is the standard deviation of the velocities $g(t_{i})$, and SNR is the signal-to-noise ratio, which we take to be 3 or 7.

We compare the performance of the adaptive V-spline with a spatially adaptive penalized spline known as the P-spline with the function asp2 from the package AdaptFitOS [29,30,31], a generalized additive model gam from the package mgcv [32,33], the kinematic interpolation approach (KI) by [16], as well as the adaptive V-spline with γ=0, which becomes a conventional spline with Hermite basis functions, and the non-adaptive V-spline where $\lambda_{0}$ is a constant. It is important to note that only the KI approach, the non-adaptive and adaptive V-splines incorporate velocity information. The V-spline parameters are obtained by minimizing the cross-validation score (22). In the gam model, we use tp basis functions with 1024 knots. For the KI approach, the position at time $t_{i}$ is interpolated from the two neighbouring points at $t_{i-1}$ and $t_{i+1}$. (The positions at $t_{1}$ and $t_{n}$ are interpolated from points at $\left(t_{1},t_{2}\right)$ and $\left(t_{n-1},t_{n}\right)$, respectively.) Following [34], we fix n=1024 in the simulations.

To examine the performance of the adaptive V-spline, we compute the true mean squared error for each of the reconstructions via:

(25) $$\mathrm{TMSE}=\frac{1}{n}\sum_{i=1}^{n}{\left(f\left(t_{i}\right)-\hat{f}\left(t_{i}\right)\right)}^{2},$$

and the Modified Nash–Sutcliffe efficiency (mNSE) [35] via:

(26) $$\mathrm{mNSE}=1-\frac{\sum_{i=1}^{n}\left|f\left(t_{i}\right)-\hat{f}\left(t_{i}\right)\right|}{\sum_{i=1}^{n}\left|f\left(t_{i}\right)-\bar{f}\right|}.$$

The results are shown in Table 1 and Table 2. The V-spline, either adaptive or non-adaptive, returns the best solution in all cases.

The reason for the poor performance of kinematic interpolation is two-fold: first, KI assumes $v_{i}$ is a good approximation to the velocity over the entire interval $\left[t_{i-1},t_{i+1}\right)$. Second, KI is not a true smoother so it is prone to errors in the observations. In contrast, the V-spline successfully smooths and interpolates in the presence of noise.

Table 3 shows the ability of the adaptive V-spline to retrieve the true SNR: for reconstruction $\hat{f}$, it is estimated by $\sigma_{\hat{f}}/\sigma_{\left(\hat{f}-y\right)}$. Table 3 shows that the estimates from the V-spline $\hat{f}$ are very close to the true values.

In summary, the simulation study has shown the ability of V-splines to accurately reconstruct trajectories from noisy and potentially problematic velocity profiles. The V-spline outperforms methods that do not use velocity information, and its smoothing strategy appears to be vastly superior to that of kinematic interpolation.

## 5. Inference of Tractor Trajectory

In this section, we apply the V-spline to a data set obtained from a GPS unit mounted on a tractor working in a horticultural setting. The motivating problem in this context is to accurately record where pesticide has been applied to ensure that neither over-spraying or under-spraying has occurred.

GPS units in vehicles provide $y_{t}$, noisy measurements of the actual position $x_{t}$, and $v_{t}$, noisy measurements of the actual velocity $u_{t}$, for a sequence of times t∈T, which is irregularly recorded with highly variable time differences $\Delta T_{i}$. These data may also be augmented with information on operating characteristics of the vehicle, $b_{t}$, in this case data on whether the tractor boom was in a raised or lowered position. The trajectory reconstruction problem is the problem of estimating $x_{s}$, for an arbitrary time *s*, given a subset of the observations $\left\{y_{t},v_{t},b_{t}|t\in T\right\}$. Note that, in this definition of trajectory reconstruction, we are not explicitly interested in estimating $u_{s}$.

The original data set consists of n=928 records of longitude, latitude, speed, bearing and the status of the tractor’s boom sprayer. The boom status, “up” and “down”, denotes the operational state of the tractor, and indicates different types of trajectories. For example, if boom status is “down”, the tractor is probably sowing, watering or harvesting on the farm. In this scenario, the speed is stable and its variance is low. On the contrary, when it is “up”, the speed could be high because the driver is travelling between jobs, it could be zero because the driver is having a break, or it might indicate the tractor is turning. In this last situation, however, the acceleration could be high. For this reason, we add further complexity to the model by allowing the penalty parameter to depend on boom status.

**Figure 2:**
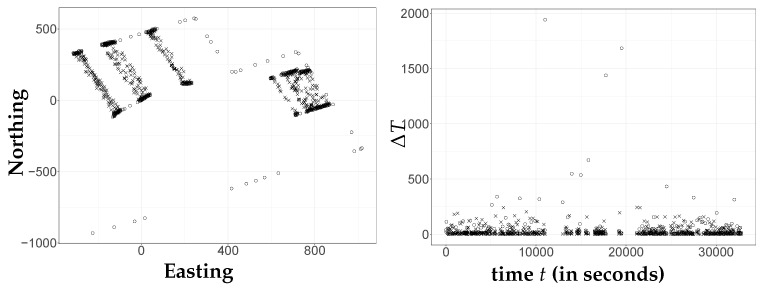
A real GPS data set of tractor movements. Observed positions in two dimensions on the left and irregular time differences indicated on the right. In trajectory reconstruction, the positions are combined with velocity information and operating characteristics of the tractor to infer actual positions for times of interest. Crosses indicate the boom is working; circles indicate it is not working.

For trajectory reconstruction, this data set was converted from *longitude* and *latitude* in degrees (${}^{\circ }$) into *easting* and *northing* in meters (*m*) by the Universal Transverse Mercator (UTM) coordinate system. The speed and bearing were converted into velocities (m/s) in those directions as well. See Figure 2.

### 5.1. The V-Spline in *d*-Dimensions

To generalize the V-spline to *d*-dimensions, we consider the situation preceding Equation (2) but where now $y_{i},v_{i}\in R^{d}$. Then, the function $f:\left[a,b\right]\to R^{d}$ is a *d*-dimensional V-spline if it minimizes:

(27) $$J\left[f\right]=\frac{1}{n}\sum_{i=1}^{n}{∥y_{i}-f\left(t_{i}\right)∥}_{d}^{2}+\frac{\gamma }{n}\sum_{i=1}^{n}{∥v_{i}-f^{\prime }\left(t_{i}\right)∥}_{d}^{2}+\int_{a}^{b}\lambda \left(t\right){∥f^{\prime \prime }\left(t\right)∥}_{d}^{2}dt,$$

where ${∥\cdot ∥}_{2}$ is the Euclidean norm in *d*-dimensions. For each direction α=1,…,d, the fitted V-spline has the form ${\hat{f}}^{\alpha }\left(t\right)=\sum_{k=1}^{2n}N_{k}\left(t\right){\hat{\theta }}_{k}^{\alpha }$, where

(28) $${\hat{\theta }}^{\alpha }={\left(B^{⊤}B+\gamma C^{⊤}C+n\Omega_{\lambda }\right)}^{-1}\left(B^{⊤}y^{\alpha }+\gamma C^{⊤}v^{\alpha }\right).$$

The parameters λ and γ are estimated by minimizing the cross-validation score:

(29) $$\underset{\lambda ,\gamma >0}{arg min}\mathrm{CV}\left(\lambda ,\gamma \right)=\underset{\lambda ,\gamma >0}{arg min}\sum_{i=1}^{n}{∥\frac{y_{i}-\hat{f}\left(t_{i}\right)+\gamma \frac{T_{ii}}{1-\gamma V_{ii}}\left(v_{i}-{\hat{f}}^{\prime }\left(t_{i}\right)\right)}{1-S_{ii}-\gamma \frac{T_{ii}}{1-\gamma V_{ii}}U_{ii}}∥}_{d}^{2}.$$

In what follows, we allow the non-adaptive and adaptive V-splines to depend on the boom status. This is to demonstrate that our method can simply and usefully also incorporate known covariates. In this application, letting $b_{i}=0$ denote boom “up”, $b_{i}=1$ denote boom “down”, and ${\bar{v}}_{i}={∥y_{i+1}-y_{i}∥}_{2}/\Delta T_{i}$ be the average velocity on the interval $\left[t_{i},t_{i+1}\right)$, the penalty term for the non-adaptive V-spline is

(30) $$\lambda_{i}=b_{i}\lambda_{d}+\left(1-b_{i}\right)\lambda_{u},$$

and, for the adaptive V-spline, it is

(31) $$\lambda_{i}=\left\{b_{i}\lambda_{d}+\left(1-b_{i}\right)\lambda_{u}\right\}\frac{\Delta T_{i}}{{\bar{v}}_{i}^{2}}.$$

Optimization in (29) is now simply with respect to positive $\lambda_{d},\lambda_{u}$ and γ.

### 5.2. Two-Dimensional Trajectory Reconstruction

**Figure 3:**
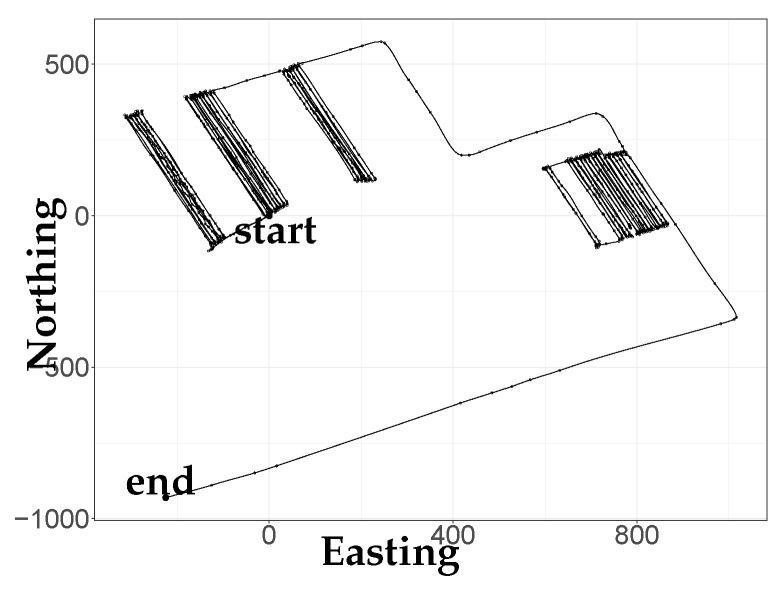
Reconstruction of the complete real GPS data set; “start” and “end” indicate the start and end points of the trajectory.

The V-spline reconstruction from the tractor data is shown in Figure 3. The parameters $\lambda_{d},\lambda_{u}$ and γ are found by our proposed cross-validation scheme using the *stats*::optim function in R [36]. It is immediately evident from the trajectory that the tractor has been moving up and down rows of an orchard or travelling between parts of the orchard.

**Figure 4:**
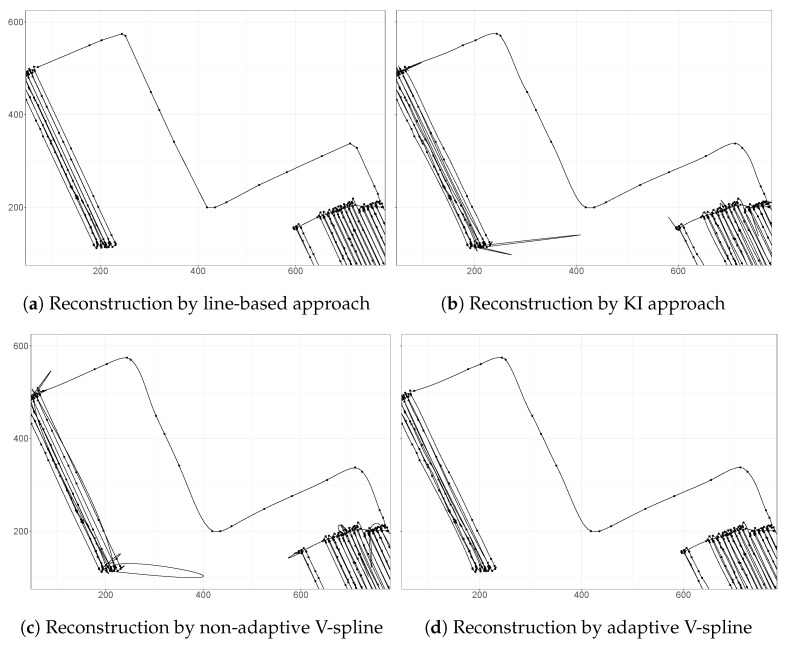
A comparison of the reconstructions by a line-based approach, kinematic interpolation, non-adaptive V-spline, and adaptive V-spline. KI relies on misleading velocity information that generates unrealistic trajectories. The non-adaptive V-spline also generates unrealistic trajectories at sharp-turning and braking points. However, the adaptive V-spline adapts to the information based on observed velocity discrepancy and generates plausible trajectories.

It is instructive to compare the performance of the adaptive V-spline to a line-based approach that simply and unrealistically connects observations by a straight line, kinematic interpolation which also utilizes velocity information, and the non-adaptive V-spline. Figure 4 shows finer detail of the tractor trajectory given by these reconstructions. A feature of the KI method is the hugely unrealistic excursions near the turn-around points at the end of each row as shown in Figure 4b. On the contrary, the adaptive V-spline, see Figure 4d, adapts to the information based on observed velocity discrepancy to avoid such excursions. Without the adaptive term (18), the non-adaptive V-spline performs in a similar way to KI, which can be seen from Figure 4c; this proves the power of the adaptive penalty.

## 6. Discussion

In this paper, a smoothing spline called the V-spline is proposed that minimizes an objective function which incorporates both position and velocity information. Given *n* knots, the V-spline has 2n effective degrees of freedom corresponding to n−1 cubic polynomials with their value and first derivative matched at the n−2 interior knots. The effective degrees of freedom are then fixed by *n* position observations and *n* velocity observations. Note that, in the limit γ→0, the V-spline reduces to having *n* effective degrees of freedom. An adaptive version of the V-spline is also introduced that seeks to control the impact of irregularly sampled observations and noisy velocity measurements.

The computational complexity of the V-spline method is equivalent to any smoothing spline that uses a cross-validation procedure to estimate the tuning parameters. The essential difference is that the V-spline incorporates 2n data points (in each dimension), as opposed to *n*. The impact of this shows up in the time to solve for $\hat{\theta }$ in (13). Thus, the computation time of the V-spline is the same as a standard smoothing spline with 2n observations. Modest computational gains can possibly be made by improving the CV parameter estimation step, but Theorem 2 already assures us that this step is highly efficient. Future research directions for the V-spline include application to ship tracking [18] and development of a fast filtering algorithm.

## Figures and Tables

**Table 1:** TMSE: * indicates the best solution (smallest error).

TMSE ($10^{-6}$)	SNR	Adpt VS	Non-Adpt VS	VS${}_{\gamma =0}$	P-Spline	gam	KI
Blocks	7	1.753 *	1.778	54.257	52.702	53.224	826.497
	3	17.036	15.339 *	152.391	145.118	154.467	4499.818
Bumps	7	1.701	1.568 *	23.436	23.447	23.446	219.259
	3	8.865 *	8.980	77.774	78.808	76.080	1193.743
HeaviSine	7	1.558 *	1.562	7.768	9.337	7.873	207.412
	3	4.360 *	8.557	33.492	34.361	33.132	1129.242
Doppler	7	1.516	0.956 *	6.668	6.406	6.435	56.910
	3	8.092 *	8.255	22.135	22.088	22.655	309.842

**Table 2:** mNSE: * indicates the best solution (closest to 1).

mNSE	SNR	Adpt VS	Non-Adpt VS	VS${}_{\gamma =0}$	P-Spline	gam	KI
Blocks	7	0.9954 *	0.9953	0.9749	0.9750	0.9752	0.9037
	3	0.9864 *	0.9870	0.9562	0.9569	0.9555	0.7753
Bumps	7	0.9917	0.9921 *	0.9700	0.9700	0.9703	0.9097
	3	0.9811 *	0.9810	0.9442	0.9428	0.9443	0.7893
HeaviSine	7	0.9915 *	0.9915	0.9820	0.9802	0.9818	0.9058
	3	0.9855 *	0.9802	0.9624	0.9617	0.9625	0.7803
Doppler	7	0.9820	0.9857 *	0.9646	0.9648	0.9646	0.8928
	3	0.9579 *	0.9575	0.9347	0.9333	0.9323	0.7499

**Table 3:** Retrieved SNR by adaptive V-spline.

SNR	True Value	*f* Known	V-Spline $\hat{f}$
*Blocks*	7	6.9442	6.9485
	3	2.9761	2.9817
*Bumps*	7	6.9442	6.9548
	3	2.9761	2.9953
*HeaviSine*	7	6.9442	6.9207
	3	2.9761	2.9891
*Doppler*	7	6.9442	6.8757
	3	2.9761	2.9372

## Data Availability

Not applicable.

## Abbreviations

The following abbreviations are used in this manuscript:

GPS	Global Positioning System
CNC	Computer (or Computerised) Numerical Control
KI	Kinematic Interpolation
RSS	Residual Sum of Squares
CV	Cross Validation
GCV	Generalised Cross Validation
TMSE	True Mean Squared Errors
mNSE	modified Nash–Sutcliffe efficiency
SNR	Signal-to-Noise Ratio
UTM	Universal Transverse Mercator

## Appendix A. Penalty Matrix in (10)

We have $\Omega_{\lambda }=\sum_{i=1}^{n-1}\lambda_{i}\Omega^{\left(i\right)}$, where ${\left[\Omega^{\left(i\right)}\right]}_{jk}=\int_{t_{i}}^{t_{i+1}}N_{j}^{\prime \prime }\left(t\right)N_{k}^{\prime \prime }\left(t\right)dt$. Thus, $\Omega^{\left(i\right)}$ is a 2n×2n bandwidth four symmetric matrix and its non-zero, upper triangular elements are

(A1) $$\begin{array}{cc} \Omega_{2i-1,2i-1}^{\left(i\right)} & =\int_{t_{i}}^{t_{i+1}}\frac{d^{2}h_{00}^{\left(i\right)}\left(t\right)}{dt^{2}}\frac{d^{2}h_{00}^{\left(i\right)}\left(t\right)}{dt^{2}}dt=\frac{12}{\Delta T_{i}^{3}} \end{array}$$

(A2) $$\begin{array}{cc} \Omega_{2i-1,2i}^{\left(i\right)} & =\int_{t_{i}}^{t_{i+1}}\frac{d^{2}h_{00}^{\left(i\right)}\left(t\right)}{dt^{2}}\frac{d^{2}h_{10}^{\left(i\right)}\left(t\right)}{dt^{2}}dt=\frac{6}{\Delta T_{i}^{2}} \end{array}$$

(A3) $$\begin{array}{cc} \Omega_{2i-1,2i+1}^{\left(i\right)} & =\int_{t_{i}}^{t_{i+1}}\frac{d^{2}h_{00}^{\left(i\right)}\left(t\right)}{dt^{2}}\frac{d^{2}h_{01}^{\left(i\right)}\left(t\right)}{dt^{2}}dt=\frac{-12}{\Delta T_{i}^{3}} \end{array}$$

(A4) $$\begin{array}{cc} \Omega_{2i-1,2i+2}^{\left(i\right)} & =\int_{t_{i}}^{t_{i+1}}\frac{d^{2}h_{00}^{\left(i\right)}\left(t\right)}{dt^{2}}\frac{d^{2}h_{11}^{\left(i\right)}\left(t\right)}{dt^{2}}dt=\frac{6}{\Delta T_{i}^{2}} \end{array}$$

(A5) $$\begin{array}{cc} \Omega_{2i,2i}^{\left(i\right)} & =\int_{t_{i}}^{t_{i+1}}\frac{d^{2}h_{10}^{\left(i\right)}\left(t\right)}{dt^{2}}\frac{d^{2}h_{10}^{\left(i\right)}\left(t\right)}{dt^{2}}dt=\frac{4}{\Delta T_{i}} \end{array}$$

(A6) $$\begin{array}{cc} \Omega_{2i,2i+1}^{\left(i\right)} & =\int_{t_{i}}^{t_{i+1}}\frac{d^{2}h_{10}^{\left(i\right)}\left(t\right)}{dt^{2}}\frac{d^{2}h_{01}^{\left(i\right)}\left(t\right)}{dt^{2}}dt=\frac{-6}{\Delta T_{i}^{2}} \end{array}$$

(A7) $$\begin{array}{cc} \Omega_{2i,2i+2}^{\left(i\right)} & =\int_{t_{i}}^{t_{i+1}}\frac{d^{2}h_{10}^{\left(i\right)}\left(t\right)}{dt^{2}}\frac{d^{2}h_{11}^{\left(i\right)}\left(t\right)}{dt^{2}}dt=\frac{2}{\Delta T_{i}} \end{array}$$

(A8) $$\begin{array}{cc} \Omega_{2i+1,2i+1}^{\left(i\right)} & =\int_{t_{i}}^{t_{i+1}}\frac{d^{2}h_{01}^{\left(i\right)}\left(t\right)}{dt^{2}}\frac{d^{2}h_{01}^{\left(i\right)}\left(t\right)}{dt^{2}}dt=\frac{12}{\Delta T_{i}^{3}} \end{array}$$

(A9) $$\begin{array}{cc} \Omega_{2i+1,2i+2}^{\left(i\right)} & =\int_{t_{i}}^{t_{i+1}}\frac{d^{2}h_{01}^{\left(i\right)}\left(t\right)}{dt^{2}}\frac{d^{2}h_{11}^{\left(i\right)}\left(t\right)}{dt^{2}}dt=\frac{-6}{\Delta T_{i}^{2}} \end{array}$$

(A10) $$\begin{array}{cc} \Omega_{2i+2,2i+2}^{\left(i\right)} & =\int_{t_{i}}^{t_{i+1}}\frac{d^{2}h_{11}^{\left(i\right)}\left(t\right)}{dt^{2}}\frac{d^{2}h_{11}^{\left(i\right)}\left(t\right)}{dt^{2}}dt=\frac{4}{\Delta T_{i}} \end{array}$$

where $\Delta T_{i}=t_{i+1}-t_{i}$ and i=1,2,…,n−1.

## Appendix B. Proof of Theorem 1

*For n≥2, the objective function J[f] is uniquely minimized by a V-spline, piecewise on the intervals $\left[t_{i},t_{i+1}\right)$, i=1,…,n−1, and linear on $\left[a,t_{1}\right]$ and $\left[t_{n},b\right]$.*

Our proof is an extension of the smoothing spline proof in [22].

**Proof.** If g:[a,b]→R is a proposed minimizer, construct a cubic spline f(t) that agrees with g(t) as well as $f^{\prime }\left(t\right)$ agrees with $g^{\prime }\left(t\right)$ at $t_{1},\ldots ,t_{n}$, and is linear on $\left[a,t_{1}\right]$ and $\left[t_{n},b\right]$. Let h(t)=g(t)−f(t). Then, for i=1,⋯,n−1,

$$\begin{array}{cc} \int_{t_{i}}^{t_{i+1}}f^{\prime \prime }\left(t\right)h^{\prime \prime }\left(t\right)dt & =f^{\prime \prime }\left(t\right)h^{\prime }\left(t\right){|}_{t_{i}}^{t_{i+1}}-\int_{t_{i}}^{t_{i+1}}f^{\prime \prime \prime }\left(t\right)h^{\prime }\left(t\right)dt\\ \; & =0-f^{\prime \prime \prime }\left(t_{i}^{+}\right)\int_{t_{i}}^{t_{i+1}}h^{\prime }\left(t\right)dt\\ \; & =-f^{\prime \prime \prime }\left(t_{i}^{+}\right)\left(h\left(t_{i+1}\right)-h\left(t_{i}\right)\right)=0. \end{array}$$

Additionally, $\int_{a}^{t_{1}}f^{\prime \prime }\left(t\right)h^{\prime \prime }\left(t\right)dt=\int_{t_{n}}^{b}f^{\prime \prime }\left(t\right)h^{\prime \prime }\left(t\right)dt=0$, since f(t) is assumed linear outside the knots. Thus, for i=0,…,n,

$$\begin{array}{cc} \; & \int_{t_{i}}^{t_{i+1}}{\left|g^{\prime \prime }\left(t\right)\right|}^{2}dt=\int_{t_{i}}^{t_{i+1}}{\left|f^{\prime \prime }\left(t\right)+h^{\prime \prime }\left(t\right)\right|}^{2}dt\\ = & \int_{t_{i}}^{t_{i+1}}{\left|f^{\prime \prime }\left(t\right)\right|}^{2}dt+2\int_{t_{i}}^{t_{i+1}}f^{\prime \prime }\left(t\right)h^{\prime \prime }\left(t\right)dt+\int_{t_{i}}^{t_{i+1}}{\left|h^{\prime \prime }\left(t\right)\right|}^{2}dt\\ = & \int_{t_{i}}^{t_{i+1}}{\left|f^{\prime \prime }\left(t\right)\right|}^{2}dt+\int_{t_{i}}^{t_{i+1}}{\left|h^{\prime \prime }\left(t\right)\right|}^{2}dt\\ \geq & \int_{t_{i}}^{t_{i+1}}{\left|f^{\prime \prime }\left(t\right)\right|}^{2}dt. \end{array}$$

The result J[f]≤J[g] follows since $\lambda_{i}>0$. Furthermore, equality of the curvature penalty term only holds if g(t)=f(t). On $\left[t_{1},t_{n}\right]$, we require $h^{\prime \prime }\left(t\right)=0$ but since $h\left(t_{i}\right)=h^{\prime }\left(t_{i}\right)=0$ for i=1,…,n, this means h(t)=0. Meanwhile on $\left[a,t_{1}\right]$ and $\left[t_{n},b\right]$, $f^{\prime \prime }\left(t\right)=0$ so that equality requires $g^{\prime \prime }\left(t\right)=0$. Since f(t) agrees with g(t) and as well as $f^{\prime }\left(t\right)$ agrees with $g^{\prime }\left(t\right)$ at $t_{1}$ and $t_{n}$, equality is forced on both intervals. □

## Appendix C. Proof of Theorem 2

*The cross-validation score of a V-spline satisfies*

$$\underset{\lambda ,\gamma >0}{arg min}CV\left(\lambda ,\gamma \right)\;=\;\underset{\lambda ,\gamma >0}{arg min}\sum_{i=1}^{n}{\left(\frac{y_{i}-\hat{f}\left(t_{i}\right)+\gamma \frac{T_{ii}}{1-\gamma V_{ii}}\left(v_{i}-{\hat{f}}^{\prime }\left(t_{i}\right)\right)}{1-S_{ii}-\gamma \frac{T_{ii}}{1-\gamma V_{ii}}U_{ii}}\right)}^{2},$$

*where $\hat{f}$ is the V-spline smoother calculated from the full data set with smoothing parameter λ and γ, and $S_{ii}={\left[S_{\lambda ,\gamma }\right]}_{ii}$, etc.*

**Proof.** We start with the following lemma: **Lemma** **A1.**
*For λ(t), γ and for fixed i, let $f^{\left(-i\right)}$ be the vector with components $f_{j}^{\left(-i\right)}={\hat{f}}^{\left(-i\right)}\left(t_{j},\lambda ,\gamma \right)$, ${f\prime }^{\left(-i\right)}$ by the vector with components ${f\prime }_{j}^{\left(-i\right)}={\hat{f}\prime }^{\left(-i\right)}\left(t_{j},\lambda ,\gamma \right)$, and define vectors $y^{*}$ be the vector of $y_{i}^{*}$ and $v^{*}$ be the vector of $v_{i}^{*}$ by*

(A11) $$\begin{array}{c} \left\{\begin{array}{cc} y_{j}^{*}=y_{j} & j\neq i\\ y_{i}^{*}={\hat{f}}^{\left(-i\right)}\left(t_{i}\right) & otherwise \end{array}\right. \end{array}$$

(A12) $$\begin{array}{c} \left\{\begin{array}{cc} v_{j}^{*}=v_{j} & j\neq i\\ v_{i}^{*}={\hat{f}\prime }^{\left(-i\right)}\left(t_{i}\right) & otherwise \end{array}\right. \end{array}$$

*Then,*

(A13) $$\begin{array}{cc} {\hat{f}}^{\left(-i\right)} & =Sy^{*}+\gamma Tv^{*} \end{array}$$

(A14) $$\begin{array}{cc} {\hat{f}\prime }^{\left(-i\right)} & =Uy^{*}+\gamma Vv^{*} \end{array}$$

**Proof of Lemma** **A1.** For any smooth curve *f* with $y^{*}$ and $v^{*}$, we have

$$\begin{array}{cc} \; & \frac{1}{n}\sum_{j=1}^{n}{\left(y_{j}^{*}-f\left(t_{j}\right)\right)}^{2}+\frac{\gamma }{n}\sum_{j=1}^{n}{\left(v_{j}^{*}-f^{\prime }\left(t_{j}\right)\right)}^{2}+\sum_{j=1}^{n}\lambda_{j}\int_{t_{j}}^{t_{j+1}}{\left(f^{\prime \prime }\left(t\right)\right)}^{2}dt\\ \geq & \frac{1}{n}\sum_{j\neq i}{\left(y_{j}^{*}-f\left(t_{j}\right)\right)}^{2}+\frac{\gamma }{n}\sum_{j\neq i}{\left(v_{j}^{*}-f^{\prime }\left(t_{j}\right)\right)}^{2}+\sum_{j=1}^{n}\lambda_{j}\int_{t_{j}}^{t_{j+1}}{\left(f^{\prime \prime }\left(t\right)\right)}^{2}dt\\ \geq & \frac{1}{n}\sum_{j\neq i}{\left(y_{j}^{*}-{\hat{f}}^{\left(-i\right)}\left(t_{j}\right)\right)}^{2}+\frac{\gamma }{n}\sum_{j\neq i}{\left(v_{j}^{*}-{\hat{f}\prime }^{\left(-i\right)}\left(t_{j}\right)\right)}^{2}+\sum_{j=1}^{n}\lambda_{j}\int_{t_{j}}^{t_{j+1}}{\left({\hat{f}}^{{}^{\prime \prime }\left(-i\right)}\left(t\right)\right)}^{2}dt\\ = & \frac{1}{n}\sum_{j=1}^{n}{\left(y_{j}^{*}-{\hat{f}}^{\left(-i\right)}\left(t_{j}\right)\right)}^{2}+\frac{\gamma }{n}\sum_{j=1}^{n}{\left(v_{j}^{*}-{\hat{f}\prime }^{\left(-i\right)}\left(t_{j}\right)\right)}^{2}+\sum_{j=1}^{n}\lambda_{j}\int_{t_{j}}^{t_{j+1}}{\left({\hat{f}}^{{}^{\prime \prime }\left(-i\right)}\left(t\right)\right)}^{2}dt. \end{array}$$

By the definition of ${\hat{f}}^{\left(-i\right)}$, ${\hat{f}\prime }^{\left(-i\right)}$ and the fact that $y_{i}^{*}={\hat{f}}^{\left(-i\right)}\left(t_{i}\right)$, $v_{i}^{*}={\hat{f}\prime }^{\left(-i\right)}\left(t_{i}\right)$. It follows that ${\hat{f}}^{\left(-i\right)}$ is the minimizer of the objective function (2), so that

$$\begin{array}{cc} {\hat{f}}^{\left(-i\right)} & =Sy^{*}+\gamma Tv^{*}\\ {\hat{f}\prime }^{\left(-i\right)} & =Uy^{*}+\gamma Vv^{*} \end{array}$$

as required.    ☐ As a consequence of lemma A1, we obtain expressions for the deleted residuals $y_{i}-{\hat{f}}^{\left(-i\right)}\left(t_{i}\right)$ and $v_{i}-{\hat{f}\prime }^{\left(-i\right)}\left(t_{i}\right)$ in terms of $y_{i}-\hat{f}\left(t_{i}\right)$ and $v_{i}-{\hat{f}}^{\prime }\left(t_{i}\right)$, respectively:

(A15) $$\begin{array}{cc} \; & {\hat{f}}^{\left(-i\right)}\left(t_{i}\right)-y_{i}=\sum_{j=1}^{n}S_{ij}y_{j}^{*}+\gamma \sum_{j=1}^{n}T_{ij}v_{j}^{*}-y_{i}\\ = & \sum_{j\neq i}^{n}S_{ij}y_{j}+\gamma \sum_{j\neq i}^{n}T_{ij}v_{j}+S_{ii}{\hat{f}}^{\left(-i\right)}\left(t_{i}\right)+\gamma T_{ii}{\hat{f}\prime }^{\left(-i\right)}\left(t_{i}\right)-y_{i}\\ = & \sum_{j=1}^{n}S_{ij}y_{j}+\gamma \sum_{j=1}^{n}T_{ij}v_{j}+S_{ii}\left({\hat{f}}^{\left(-i\right)}\left(t_{i}\right)-y_{i}\right)+\gamma T_{ii}\left({\hat{f}\prime }^{\left(-i\right)}\left(t_{i}\right)-v_{i}\right)-y_{i}\\ = & \left(\hat{f}\left(t_{i}\right)-y_{i}\right)+S_{ii}\left({\hat{f}}^{\left(-i\right)}\left(t_{i}\right)-y_{i}\right)+\gamma T_{ii}\left({\hat{f}\prime }^{\left(-i\right)}\left(t_{i}\right)-v_{i}\right) \end{array}$$

and

(A16) $$\begin{array}{cc} \; & {\hat{f}\prime }^{\left(-i\right)}\left(t_{i}\right)-v_{i}=\sum_{j=1}^{n}U_{ij}y_{j}^{*}+\gamma \sum_{j=1}^{n}V_{ij}v_{j}^{*}-v_{i}\\ = & \sum_{j\neq i}^{n}U_{ij}y_{j}+\gamma \sum_{j\neq i}^{n}V_{ij}v_{j}+U_{ii}{\hat{f}}^{\left(-i\right)}\left(t_{i}\right)+\gamma V_{ii}{\hat{f}\prime }^{\left(-i\right)}\left(t_{i}\right)-v_{i}\\ = & \sum_{j=1}^{n}U_{ij}y_{j}+\gamma \sum_{j=1}^{n}V_{ij}v_{j}+U_{ii}\left({\hat{f}}^{\left(-i\right)}\left(t_{i}\right)-y_{i}\right)+\gamma V_{ii}\left({\hat{f}\prime }^{\left(-i\right)}\left(t_{i}\right)-v_{i}\right)-v_{i}\\ = & \left({\hat{f}}^{\prime }\left(t_{i}\right)-v_{i}\right)+U_{ii}\left({\hat{f}}^{\left(-i\right)}\left(t_{i}\right)-y_{i}\right)+\gamma V_{ii}\left({\hat{f}\prime }^{\left(-i\right)}\left(t_{i}\right)-v_{i}\right). \end{array}$$

Thus,

(A17) $${\hat{f}\prime }^{\left(-i\right)}\left(t_{i}\right)-v_{i}=\frac{{\hat{f}}^{\prime }\left(t_{i}\right)-v_{i}}{1-\gamma V_{ii}}+\frac{U_{ii}\left({\hat{f}}^{\left(-i\right)}\left(t_{i}\right)-y_{i}\right)}{1-\gamma V_{ii}}.$$

By substituting Equation (A17) into (A15), we obtain

$${\hat{f}}^{\left(-i\right)}\left(t_{i}\right)-y_{i}=\frac{\hat{f}\left(t_{i}\right)-y_{i}+\gamma \frac{T_{ii}}{1-\gamma V_{ii}}\left({\hat{f}}^{\prime }\left(t_{i}\right)-v_{i}\right)}{1-S_{ii}-\gamma \frac{T_{ii}}{1-\gamma V_{ii}}U_{ii}}.$$

Consequently,

$$\underset{\lambda ,\gamma >0}{arg min}CV\left(\lambda ,\gamma \right)=\underset{\lambda ,\gamma >0}{arg min}\sum_{i=1}^{n}{\left(\frac{\hat{f}\left(t_{i}\right)-y_{i}+\gamma \frac{T_{ii}}{1-\gamma V_{ii}}\left({\hat{f}}^{\prime }\left(t_{i}\right)-v_{i}\right)}{1-S_{ii}-\gamma \frac{T_{ii}}{1-\gamma V_{ii}}U_{ii}}\right)}^{2}.$$

    ☐
